# Spontaneous Ad Hoc Mobile Cloud Computing Network

**DOI:** 10.1155/2014/232419

**Published:** 2014-08-17

**Authors:** Raquel Lacuesta, Jaime Lloret, Sandra Sendra, Lourdes Peñalver

**Affiliations:** ^1^Universidad San Jorge, A-23, km 299, Villanueva de Gállego, 50830 Zaragoza, Spain; ^2^Universidad Politécnica de Valencia, Camino Vera S/N, 46022 Valencia, Spain

## Abstract

Cloud computing helps users and companies to share computing resources instead of having local servers or personal devices to handle the applications. Smart devices are becoming one of the main information processing devices. Their computing features are reaching levels that let them create a mobile cloud computing network. But sometimes they are not able to create it and collaborate actively in the cloud because it is difficult for them to build easily a spontaneous network and configure its parameters. For this reason, in this paper, we are going to present the design and deployment of a spontaneous ad hoc mobile cloud computing network. In order to perform it, we have developed a trusted algorithm that is able to manage the activity of the nodes when they join and leave the network. The paper shows the network procedures and classes that have been designed. Our simulation results using Castalia show that our proposal presents a good efficiency and network performance even by using high number of nodes.

## 1. Introduction

A mobile ad hoc network (MANET) is a self-configuring network of mobile devices connected by wireless links. Each device in a MANET is free to move independently in any direction and will therefore frequently change its links to other devices. Each device must forward traffic unrelated to its own use and therefore could act as a router.

A spontaneous ad hoc network is a type of ad hoc network that is formed during a certain period of time, with no dependence on a central server and without the intervention of an expert user [1]. This network is made of several independent nodes which are in the same place at the same time in order to communicate with each other. Nodes are free to join and leave the network at will [2]. Spontaneous networking happens when neighboring nodes discover each other within a short period of time; however, discovery velocity is paid in terms of energy consumption [3]. Spontaneous networks are conceptually in a higher level of abstraction than ad hoc ones; they are basically those who seek to imitate human relationships in order to work together in groups, running on the already existing technology. Their objective is the integration of services and devices in an environment which allows the provision to the user of an instant service with minimum manual intervention, ensuring important aspects, such as the multimedia quality [4] or network lifetime [5]. The concept of spontaneous networks was introduced in depth by Feeney et al. in [6].

The biggest problem in these networks is the security issue [7, 8]. The use of a certificate authority (CA) server is not a good idea because of the lack of a robust infrastructure and the distance. The device could be very far from the CA, so their connection could be a big issue. There is a need of a two-phase protocol to allow the exchange of an introductory description of each device, that is, a handshake of the devices within the same area. In order to achieve this, a protocol where each device has to exchange an identity card and will have a neighbour card list has been proposed. Thus, each node takes over the role of the CA. The model uses the trust between nodes as a key base of the proposed protocol. Castalia simulator has been used in order to validate our proposed spontaneous network.

The remainder of this paper is structured as follows.  Section 2 shows some previous works about spontaneous ad hoc networks and mobile cloud computing. The proposed spontaneous ad hoc network model for mobile computing is explained in Section 3. It details the analytical model and the cryptographic and the trust system for protecting the mobile cloud network.  Section 4 explains the designed node algorithms, the network procedures, and the designed classes. The network performance is shown in Section 5. The deployment is explained in section. Finally, Section 7 shows the conclusion and future work.

## 2. Related Work

In mobile computing, there are some inherent problems such as resource scarcity, frequent disconnections, and mobility that make exploiting its full potential difficult. In [9], authors propose to address these problems by executing mobile applications on resource providers external to the mobile device. They provide an extensive survey of mobile cloud computing research highlighting the motivation for mobile cloud computing as the dominant model for mobile applications in the future. On mobile cloud computing, devices can act as clients or resource providers. Some requirements such as adaptability, scalability, availability, and self-awareness need to be met in a cloud. They present a taxonomy of the issues found in this area and the approaches in which these issues have been tackled. They focused their study on operational level, end user level, service and application level, and security and context-awareness level. They remark that although many of the reviewed frameworks mention the need for security and trust, very few of them have actually implemented it; they have left the implementation for future directions.

In this kind of network, it is very important to determine the application in which the network will be dedicated to. As a function of this, factors such as network size, the type of devices, the software applications, and the shared services will be defined. Another important issue is the routing protocol used to communicate all nodes. The routing protocols used in spontaneous ad hoc and sensor wireless networks could be the same as regular ones, so we should consider the same constraints such as transmission power, energy resources, bandwidth usage, delay, hop count, and QoS, among others [10]. All of these factors will be affected by issues such as link stability or level of mobility in nodes [11], which, somehow, will depend on the environment where the network is deployed.

Researchers claim that spontaneous networking presents the need of improving the wide-scale applications fully exploiting its potential and that this is due to the intrinsic complexity of spontaneous network management, unsuitable to be directly handled by application developers. Following this approach, Bellavista et al. [12] proposed a middleware called RAMP for managing the autonomic and cross-layer application of spontaneous networks. The RAMP prototype can be considered as a useful tool for the community of researchers in the field of generation of spontaneous networks. RAMP enables the dynamic sharing of all resources available via multiple, heterogeneous, intermittent, infrastructure-based, and ad hoc links, which are orchestrated in a lightweight way to compose the multihop paths needed to share applications at runtime. RAMP performance is evaluated considering aspects such as delay requirements in real-time multimedia streaming. Finally, the simulation results show that the feasibility of this proposal achieves good results in a wide range of practical situations with different file sizes and path lengths.

Christensen [13] examines the architectural considerations of creating next generation mobile applications using smart mobile devices, context enablement using sensors on the device and cloud computing and RESTful web services. Mobile applications are enhanced with REST based cloud computing technologies to create applications on the smart mobile device with offload processing. To best leverage this, they consider the capabilities and constraints of these architectures.

Mani et al. presented in [14] a platform called SCOPE that implements an architecture to provide a P2P and spontaneous solution for social networking in local areas. SCOPE follows the hierarchical P2P model because in a network there are nodes with higher computing capability which can form an overlay and provide the distributed data management system for the P2P social network, meanwhile, client nodes connect to supernodes and rely on them for sharing their contents or accessing to the shared information. SCOPE is based on IEEE 802.11 ad hoc mode and needs no infrastructure. SCOPE is developed to work on mobile devices spontaneously without any dedicated network resources. The proposal provides the distributed database and lookup services deployed on distributed hash table (DHT) technology where it defines the rules for information management and creates our social networking overlay. As authors conclude, this proposal is able to provide session based communication services and provides a rich menu of social networking services from simple link/text sharing to P2P IP telephony.

There are lots of applications where spontaneous networks can be very useful. Regarding the environmental monitoring, Liu et al. presented an adaptive and efficient peer-to-peer search (AEPS) approach for distributed service discovery for dependable service integration on service-oriented architecture [15]. The proposal is able to efficiently discover desirable services for decision making of disaster monitoring and relief by interacting with connected nodes with incomplete information. AEPS builds a social network for each sensor node which contributes to an effective service discovery. AEPS is evaluated for rescue capability provision where the results demonstrated that distributed nodes can self-organize in a peer-to-peer way and discover the required service and information without any central administration. In this case, the creation of a spontaneous network is used to assist an emergency rescue team to make the correct real-time decisions in very changing environments.

In [16], the authors studied how the underused computing resources within an enterprise may be harnessed to improve their utilization and create an elastic computing infrastructure. They propose to use an ad hoc cloud model that allows complex cloud-style applications to exploit untapped resources on nondedicated hardware. They have outlined a case for ad hoc cloud computing, a set of resulting research challenges, and they propose an architecture. No protocol is designed and developed in this paper.

Taking into account that mobile devices are resource-constrained and some applications demand more resources than they can afford, in [17], Huerta-Canepa and Lee propose to create a virtual cloud computing platform using mobile phones. They present the preliminary design of a framework to create ad hoc cloud computing providers. The framework creates a cloud among the devices in the vicinity, allowing them to execute jobs between the devices. However, the work presented is preliminary, and no protocol has neither been designed nor detailed.

Another solution is the presented by Divya in [18]. He proposes a conceptual architecture where a mobile application platform shares a service among multiple users. A proof-of-concept prototype is developed using Android. The server platform shares Android OS among multiple users to obtain high performance on virtual image-based virtualization for mobile applications.

In this sense, several authors of this paper have previously deployed a spontaneous ad hoc network for multiple purposes, but never for cloud computing. In [19], a secure protocol for spontaneous wireless ad hoc networks was proposed. It is based on the behavior of human relationships. It uses a hybrid symmetric/asymmetric scheme and the trust between users in order to exchange the initial data and to exchange the secret keys that will be used to encrypt the data. In this paper, authors explained the procedures for the node's self-configuration and for providing DNS service. In [20], two flexible secure spontaneous wireless ad hoc network protocols for wireless mesh clients that are based on the computational costs are proposed. Proposals are based on a trust network, where the session key allows node confidentiality. They have been implemented over the DSR routing protocol. The developed protocols provide node authenticity and intermediate node authenticity when packets are transmitted. Integrity checking, random checking, and verification distribution are also considered in the protocol. In [21], we propose a secure spontaneous ad hoc network, based on direct peer-to-peer interaction, to grant a quick, easy, and secure access to the users when they surf the web. The protocol allows the users to collaborate during a period of time to accomplish a collaborative task. The proposal is also compared with other caching techniques published in the related literature. The proposed solution presents a distributed model where the interaction required between devices is minimal. In [22], we proposed a secure spontaneous network to create communities. Each community has an identity that acts as a unity on a world based on internet connection. Trust chains are established among users. Chains of confidence allow the establishment of groups or communities to access the services as well as for spreading group information.

As we have seen, no previous spontaneous network has been focused on mobile cloud computing.

## 3. Network Model and Description

In this section, the proposed spontaneous ad hoc network for mobile cloud computing and its model is described.

Our network model meets the following requirements.

1. Devices can move freely in the given area. Even out of each other's range.
2. Every node is also a router. It has a limited communication range towards other nodes.
3. The different identities are given by IP addresses. Each address is obtained dynamically following our previous proposal [23].
4. There is no central administration.
5. Devices can come from everywhere and join and leave at will.
6. Resources for cloud computing can be provided by any node if it has enough capacity to do it.

During the start-up of a node, it broadcasts messages in order to find neighbors. In this kind of networks, it is very important to select those nodes which offer better performance to the whole network [24]. A node will accept another node as its neighbor as a function of the amount of messages it has received to this node. When a new node has defined all its neighbors, it sends its identity card to all its neighbors. If the neighbor sends back a message to inform that it has received the identity card and the content of this message is correct (by checking the hash of the message), then the new node trusts these neighbors. When a node trusts a second node, it can send messages directly to the second node, unless the second node does not trust the first node; in this case, the communication is not allowed. The system does not follow the commutative and associative properties; that is, although a first node can trust a second node, the second node may not trust the first node. It also happens with three nodes in a chain. If a node wants to send a message towards a nontrusted node, then it has to do it through a trusted node.

The system follows the next steps.

1. Broadcast messages searching neighbor nodes.
2. Send its identity card to the neighbors.
3. Acknowledge/not acknowledge the reception of the messages from its neighbors.
4. Set the neighbor node as a trusted or nontrusted node.

Our protocol is based on the use of two information structures: (1) an IDC (identity card) and (2) a certificate. On one hand, the IDC is composed by two parts. The first one is the public part which is formed by a logical identity (LID). It is unique for each user and allows nodes to identify it. LID includes information such as name, photograph, and other user identification. Public part also contains information about the public key of the user (*K*_*i*_) and the information signature. On the other hand, private part is composed by the private key (*k*_*i*_) and this information is not accessible by other devices.

A certificate of a user consists of a validated identification card, signed by the user that gives its validity, for example, user “*j*”. Thus, the certificate of user “*i*”, validated and signed by the user “*j*”, corresponds to “*C*_*ij*_”. The user will introduce its LID only the first time that the user uses the system because the security information is generated only the first time the user joins the network. Security data is stored persistently in the device for its future use.

In order to explain the procedure, the following case is detailed. When there are three persons and two of them know certain data, then there is only one way that lets the third person know this data: one of the two persons must trust the third person. This simple example explains basically the concept of trusted network and how data are exchanged between nodes.

If the ad hoc network covers a large area, then the cloud computing services can be obtained by using ad hoc routing. In this proposal, the ad hoc routing is performed only between trusted nodes, so there are two important facts: (1) nodes should not be trusted without the proper authentication of the node and user and (2) confidentiality, integrity, availability, and access control with authentication, all of them must be based on encryption mechanisms that must be offered without central administration.

If we want to create a spontaneous ad hoc network for mobile cloud computing, we need trust establishment, key management, and membership control. Network availability and routing security must also be added. Techniques that enable the creation of ad hoc networks based on the spontaneity of human interactions (people who are near each other can communicate, exchange things, and ask people to relay information to others) should be added. In our model, each node will send its public key towards its neighbors. When the node obtains a public key, it is considered valid only if it is sure that it belongs to the owner. Not valid means that it is not sure that the key belongs to the owner. If the node trusts the key, it signs the key with its private key and considers the node as a trusted neighbor. Then, a trust network is created. When a new device joins the network and it does not have a pair of keys, it must generate them to perform authentication and to communicate with other nodes. When a node leaves the network, the network maintains the data for a period of time in case it wants to come back later. But it has to authenticate again. A node does not have to obtain the public key from every other node; in other words, one node does not have to broadcast its authentication information to all other nodes in the network. Nodes can obtain this information through the “network of trust.” Now, we provide a simple example where the network is formed by three nodes. Node 1 and node 2 know and trust each other. Then, node 2 trusts a third node, node 3. If the second node receives a public key from the third node and signs it with its private key, we consider that the owner of this key is “trusted.” Later, if the first node wants to obtain that key, it can be obtained from the second node, and since the first node trusts the second one, it “validates” this new key by signing it with its private key. If the third node is not trustworthy, any key signed by the third node will not be considered a trusted key. Furthermore, the first node will never sign the third node key, although it might forward it to other nodes in the spontaneous ad hoc mobile cloud computing network.

### 3.1. Analytical Considerations

In this section, we analyze our proposal analytically. Our purpose is to model the behavior of the spontaneous ad hoc network when there are nodes joining and leaving during its existence. On one hand, we will take care of the authentication and trust between nodes (by using trust links and trusted communication graphs), and, on the other hand, we model the network behavior (in terms of number of nodes in the network because of leavings and joining of new nodes) by using the conditional probability density function. Let *N*(*t*) be a set of users having a meeting with wireless devices, with |*N*(*t*)| = *n* being the maximum number of users during a certain period of time *t*. These users are located in a certain bounded region *R*. The trusted communication graph is the directed graph *G*(*t*) = (*N*(*t*), *E*(*t*)) such that each pair of users (*u*, *v*) ∈ *E*(*t*) only if the user's device *v* is within *u*'s transmitting range at the current transmit power level at time *t*. The graph contains all possible wireless links between the nodes in the network. Given any two nodes *u*, *w* ∈ *N*, a path connecting *u* and *w* in *G* is a sequence of nodes {*u* = *u*_0_, *u*_1_,…, *u*_*k*−1_, *u*_*k*_ = *v*} such that for any *i* = 0,…, *k* − 1, (*u*_*i*_, *u*_*i*+1_) ∈ *E*. The length of the path is the number of edges in the path. Moreover, *u* has a pair of values (*T*, *V*) for each node *w* which gives the trust (*T*) and validity (*V*) values for each user. The trust and validity can only have two values: *T* = {0,1} and *V* = {0,1}.

Users can join and leave the spontaneous network at will, so a range assignment RA is said to be connected at time *t*, if the resulting communication graph at time *t* is strongly connected; that is, if for any pair of nodes *u* and *v*, there exists at least one trusted connection from *u* to *v*. In other words, the trusted directed wireless link (*u*, *v*) exists if and only if nodes *u* and *v* are at distance of at most RA(*u*) at time *t* and their* trusted* parameter value is equal to 1. In this case, *v* is said to be a 1-hop neighbor, or* neighbor* for short, of node *u*. The trust nodes set of node *u*, denoted as TNS(*u*), is defined as it is shown in the following expression:

(1) $$\begin{array}{c} \text{TNS}\left(u\right)=\left\{z\in N:\left(z,u\right)\in E,T=1\right\}. \end{array}$$

A trusted wireless link is said to be bidirectional, or symmetric, at time *t* if (*u*, *z*) ∈ *E*(*t*), (*z*, *u*) ∈ *E*(*t*), *u* trusts *z*, and *z* trusts *u*. The trusted communication graph generated can be considered as undirected, since (*u*, *z*) ∈ *E*(*t*)⇔(*z*, *u*) ∈ *E*(*t*).

Let us suppose that a user *u* has authenticated the user *w* and *u* has *w*'s public key, then *u* sends a message encrypted with the session key to *w*. In this case, we say that there is a trusted directed graph from *u* to *w*. For any trusted directed graph *H* ∈ *G*, if two users *u* and *w* are in *H*, and there is a trusted directed path from *u* to *w* in *H*, then we say that *w* is reachable from *u* in *H* and we denote this by (*u*↔*w*)_*H*_; thus, *w* is also reachable from *u* in *G*, and we denote this by (*u*↔*w*)_*G*_.

In order to use a public key distribution system for user authentication and session key sharing, each user maintains a local repository of public key certificates and their* trust* values. When the user *u* wants to use the resources shared by user *w*, first *w* must trust *u*, so they must merge their subgraphs and try to find a trusted directed path from *u* to *w*. Thus, we can apply a subgraph theory based similar to work performed by Capkun et al. in [25]. But in that case they merged subgraphs to authenticate a public key in trusted authorities or certificate repositories, not trusted paths, while we merge subgraphs to validate trusted paths.

We assume that each user has the same subgraph selection algorithm *A* to build its subgraph. We denote *S*_*A*_(*H*, *u*) by the algorithm *A* executed in *H* ∈ *G* by the user *u*. When we merge the subgraph *S*_*A*_(*H*, *u*) of user *u* with *S*_*A*_(*H*, *v*) of user *v*, we obtain *S*_*A*_(*H*, *u*, *v*). When the trusted communication graph is undirected, *S*_*A*_(*H*, *u*, *v*) = *S*_*A*_(*H*, *v*, *u*).

The performance of the subgraph selection algorithm, denoted by *P*_*A*_(*H*), is defined as the ratio of the number of user pairs (*u*, *w*), where there is a trusted directed path from *u* to *w* in the merge subgraph of *u* and *w* to the number of user pairs (*u*, *w*), where there is a directed path from *u* to *w* in the trust graph. The following expression shows *P*_*A*_(*H*)

(2) $$\begin{array}{c} P_{A}\left(H\right)=\frac{\text{Card}\left\{\left(u,v\right)\in NxN:{\left(u↔v\right)}_{S_{A}(G,u,v)}\right\}}{\text{Card}\left\{\left(u,v\right)\in NxN:{\left(u↔v\right)}_{G}\right\}}, \end{array}$$

where* card* denotes the cardinality of a set. The performance of *A* can be increased by selecting larger subgraphs, that is, using more information about the trust graph, but the devices of the users will need more memory to store their subgraphs. Moreover, the devices will need high amount of knowledge to execute it.

In order to model the network behavior when the users join the network during the meeting time, we have used the diffusion approximation. In the spontaneous network, there will be users that join and leave the ad hoc network at will. Let *t*_*i*_ be the arrival time of the user *i* to the network, and let *t*_*i*_′ be the departure time of the user *i*. That is, 0 ≤ *t*_*i*_ < *t*_*i*_′ ≤ *T*, where *T* is the network lifetime. Let *A*(*t*) and *D*(*t*) represent the cumulative number of arrivals and departures, respectively, up to time *t*. The number of users in the spontaneous network at time *t*, *N*(*t*), is given by the following expression:

(3) $$\begin{array}{c} N\left(t\right)=A\left(t\right)-D\left(t\right). \end{array}$$

Let the consecutive interarrival time *a*_*i*_ = *t*_*i*_ − *t*_*i*−1_ and the consecutive interdeparture time *d*_*i*_ = *t*_*i*_′ − *t*_*i*−1_′ be both independent and identically distributed with the (mean, variance) given by (1/*μ*_*a*_, *σ*_*a*_^2^) and (1/*μ*_*d*_, *σ*_*d*_^2^), respectively. Let their squared coefficients of variation be *C*_*a*_^2^ = *σ*_*a*_^2^ · *μ*_*a*_ and *C*_*d*_^2^ = *σ*_*d*_^2^ · *μ*_*d*_, respectively. We define the sum of a set of consecutive interarrival times as *T*_*k*_ = ∑_*i*=1_^*K*^ *a*_*i*_. We assume that they are independent and identically distributed random variables; hence, according to the central limit theorem, the standardized random variable, *T*_k_*, shown in expression (4) tends to a standard normal distribution with *k* → *∞*, as it is shown in the following expression:

(4) $$\begin{array}{c} T_{k}^{*}=\frac{T_{k}-k\cdot \mu_{a}}{\sigma_{a}\sqrt{k}}, \end{array}$$

(5) $$\begin{array}{c} N\left(t\right)=\underset{k\to \infty }{\lim}P\left[\frac{T_{k}-k\cdot \mu_{a}}{\sigma_{a}\sqrt{k}}\leq n\right]=\frac{1}{\sqrt{2\pi }}\overset{n}{\underset{-\infty }{\int }}e^{-(t^{2}/2)}dt. \end{array}$$

If *k* is large enough, there will be many arriving users between *t* and *t* + *k* and may be approximated by the normal distribution with mean *μ*_*a*_ *t* and variance *σ*_*a*_^2^ *μ*_*a*_^3^ *t*. Similarily, the number of leaving users during that time will be approximately normally distributed with mean *μ*_*d*_ *t* and variance *σ*_*d*_^2^ *μ*_*d*_^3^ *t*. Consequently, the changes of *N*(*t*) within the interval [*t*, *t* + *k*], then *N*(*t* + *k*) − *N*(*t*), should be approximately normally distributed with the mean as is shown in the following expression:

(6) $$\begin{array}{c} \beta =\left(\mu_{a}-\mu_{d}\right)t. \end{array}$$

And the variance is given by the following expression:

(7) $$\begin{array}{c} \alpha =\left(\sigma_{a}^{2}\mu_{a}^{3}+\sigma_{d}^{2}\mu_{d}^{3}\right)t. \end{array}$$

The diffusion approximation replaces *N*(*t*) by a continuous diffusion process (also known as Wiener-Levy process) *x*(*t*), normally distributed with the mean *β* · *dt* and variance *α* · *dt*. Given the initial value *x*_0_ = 0, the unrestricted process *x*(*t*) would have the conditional probability density function at time *t* given by the following expression:

(8) $$\begin{array}{c} P\left(x,t\right)=\frac{1}{\sqrt{2\pi \alpha t}}e^{-({\left(x-\beta t\right)}^{2}/2\alpha t)}, \end{array}$$

which satisfies Kolmogorov diffusion equation (also known as Fokker-Planck equation) given in the following expression:

(9) $$\begin{array}{c} \frac{\partial f\left(x,t\right)}{\partial t}=-\beta \frac{\partial f\left(x,t\right)}{\partial x}+\frac{\alpha }{2}\frac{\partial^{2}f\left(x,t\right)}{\partial x^{2}}. \end{array}$$

Deriving expression (8) in expression (9) and treating *x* = 0 as a reflecting barrier for all *t* > 0, we obtain the following expression:

(10) $$\begin{array}{c} \underset{x\to \infty }{\lim}\left[-\beta P\left(x,t\right)+\frac{\alpha }{2}\frac{\partial P\left(x,t\right)}{\partial x}\right]=0. \end{array}$$

Now, we can estimate the solution when *t* → *∞* and *μ*_*a*_ < *μ*_*d*_. Expression (11) shows the equilibrium distribution of the conditional probability density function [26]

(11) $$\begin{array}{c} P\left(x\right)=\frac{2\left|\beta \right|}{\alpha }e^{-(2|\beta |x/\alpha )}, \end{array}$$

where *α* and *β* are defined in (6) and (7), which are related to the probability of nodes' interarrival and interdeparture times.

### 3.2. Cryptographic System

The cryptographic algorithm election has been taken bearing in mind their strong security and simple key management features. Symmetric algorithms and summary functions have lower computational cost than public key cryptography, but public key cryptography has stronger security and it could be feasible in devices with low computation capacity.

In our proposal, we use a summary function with symmetric and asymmetric algorithms with the purpose of taking their benefits. The security management is based on the public key infrastructure and the symmetric key encryption scheme. Asymmetric key encryption scheme is mainly used in the distribution of session key and in the user authentication process. It lets us also generate a distributed certification authority. The symmetric key is used as a session key to cipher the confidential messages between trust nodes, because it has less energy requirements [27–29]. Asymmetric key encryption scheme is used to authenticate the users. The hybrid symmetric/asymmetric scheme is introduced to exchange the initial data and to exchange the secret keys that will be used to encrypt the data. The hash function lets us improve the data integrity. Now, we are going to discuss which algorithms are the best for our purpose.

We have used advanced encryption standard (AES) algorithm for the symmetric encryption scheme [30]. It presents a high security level because its design structure removes subkey symmetry. It is also resistant to lineal and differential cryptanalysis. AES is actually considered as one of the most secured ones. Moreover, the execution times and the energy consumption in the cryptography processes are adequate for low power devices.

The asymmetric encryption scheme should overload the devices as less as possible. On one hand, elliptic curve cryptosystem (ECC) is presented as a high performance scheme that is recommended by many researchers [31]. On the other hand Rivest, Shamir and Adleman cryptographic algorithm (RSA) is very secure and it has been checked and recommended by many scientists [32]. ECC needs fewer bits than RSA (163 bits in ECC versus 1024 bits in RSA) in order to obtain the same security level, so it is able to achieve high security level with low size keys without consuming too much system resources, thus needing less bandwidth. ECC is usually adequate for small devices with few memory resources and low computing resources (such as cellular phones and smart cards). In order to have flexibility in our protocol and because both cryptographic algorithms have good performance, we have included both (RSA and ECC) in our protocol. The election of one of them will be taken in the network formation. Both algorithms will be shown later in our performance study.

We have selected secure hash algorithm (SHA-1) for the summary function [33]. SHA-1 is commonly used because of its equilibrium between its speed and its security. This performance is also maintained in low computing devices. This feature does not happen in other functions, because they mainly depend on the processor. Its execution time and its energy consumption are not so high when they are compared with other functions.

### 3.3. Trusted Network

The proposed model is based on the creation and management of a trusted network. A node will trust other nodes through personal view and criteria. That is, the trust is based on the relationship of the users rather than on a central certification authority. The user of the device will identify the other users and will be in charge of establishing a trust value (0 or 1) associated with each one of them. The parameters used for configuring this trust network are* trust* and* validity*.

Trust refers to the person who owns the key and its value will be established by the relationship between the user that grants it and the user that is granted. It should be granted to reliable persons when their IDCs are exchanged. The* trust* can always be changed manually by the user later.* Validity* indicates that a certificate belongs to that person/device.  Table 1 shows the* trust* and* validity* values.

### 3.4. Certification Authority

**Figure 1:**
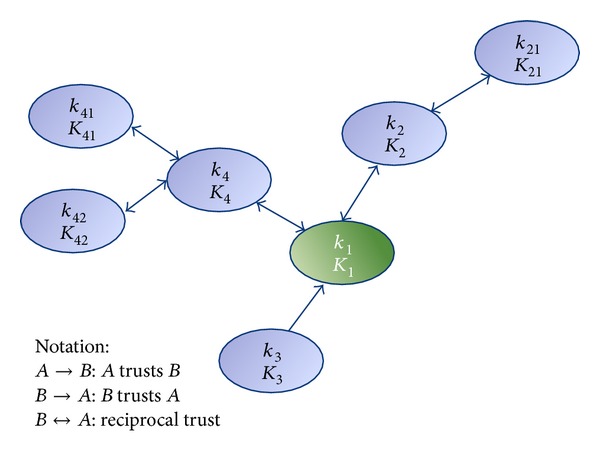
Example of trust nodes.

The certification authority of a node could be any node in the group of nodes that this node trusts. This system lets us build a distributed certification authority between trust nodes. When a node wants to communicate with other nodes and see if it is a valid node, it can request the certificate of that node to its trust nodes. After obtaining this certificate, it will be able to sign this node as a valid node. All nodes can be both, client, requesting information or authentication to other nodes, and server, serving requests for information or authentication from other nodes.  Figure 1 shows an example. Each *n* node has its public key (*K*_*n*_) and it private key (*k*_*n*_). Nodes 2 and 4 are trust nodes of node 1, but not of node 3. Thus, nodes 2 and 4 could act as a certification authority of node 1.

## 4. System Design

In this section, we explain the designed algorithms for the nodes, the network procedures, and the classes designed for Castalia in order to simulate it.

### 4.1. Network Procedures

After defining the network model and the security features that our proposed spontaneous ad hoc network should present, it is important to specify how a node should work and the set of actions it should perform to ensure the correct operation of the whole network. This subsection explains the operation of the network and the main processes included.

In order to design the flow chart diagrams, we have used the Unified Modeling Language (UML) [34]. UML is an industry standard modeling language with a rich graphical notation and comprehensive set of diagrams and elements that can be used to model object oriented systems.

#### 4.1.1. Procedure to Request the Update to all Network Nodes

**Figure 2:**
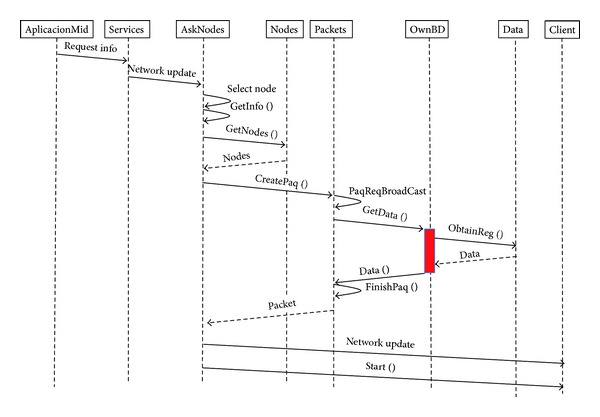
Procedure to request an update from all network nodes.

A user requests a data updating from all nodes. Firstly, the information about network nodes is obtained. Secondly, data updating packet is prepared and the request is sent.  Figure 2 shows the procedure and the primitives and services offered and served by the nodes.

#### 4.1.2. Procedure to Process a Request

**Figure 3:**
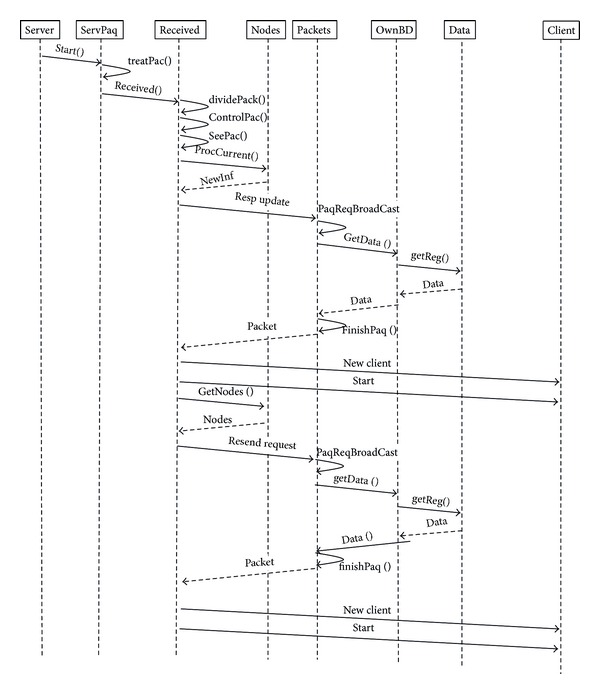
Procedure to process a request.

When a request is received, the information requested is checked and a reply is sent to the source node that sent the request. Then, the request is forwarded to the rest of network nodes. After receiving the data, request form a new client can be attended. The process to attend each request is shown in Figure 3. As the diagram shows, each task is validated by the replying of packets with data information, node information, or control packets. After the confirmation, a new packet is sent in order to complete the communication between nodes.

### 4.2. Node Algorithms

#### 4.2.1. Packet Control

**Figure 4:**
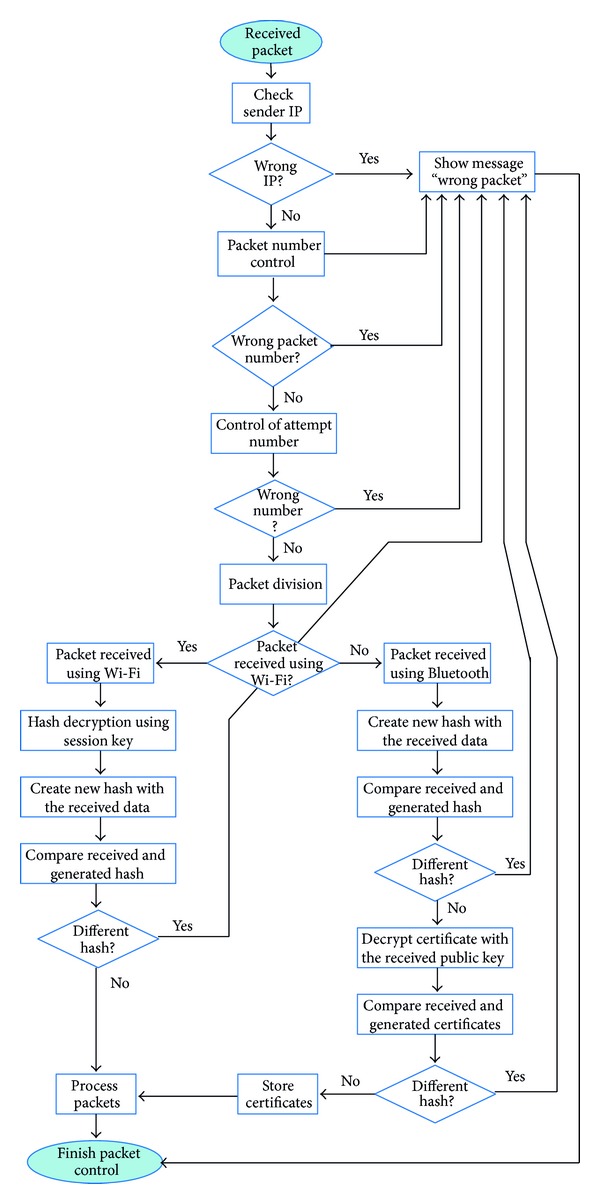
Packet control.

When a packet is received by a node, it applies a packet control process to the received data.  Figure 4 shows it. Data are checked to know whether it is correct and it has not been modified during the transmission or not. The source IP, packet number, and retry number are some of the checked parameters. This check is performed just for security reasons, despite of its analysis at lower levels such as the underlying wireless communication technology (Bluetooth, Wi-Fi, etc.).

When Bluetooth is used, the Bluetooth frames are used in the authentication process. In this case, the packet digest (hash) is not ciphered with the session key because the receiver node does not have this key yet. The packet includes the sender's node certificate ciphered with the sender's private key. Sender's public key is also included in the packet. Sender's node certificate is deciphered with its public key.

When Wi-Fi is used, the packet digest is ciphered with the session key. When the frame is received, data are firstly deciphered and then the frame is checked.

In both cases, if the hash comparison is wrong, the system shows a message, informing that the frame is wrong. If the results of the comparisons are valid, packets are processed and this process ends.

#### 4.2.2. Modification of Keys

**Figure 5:**
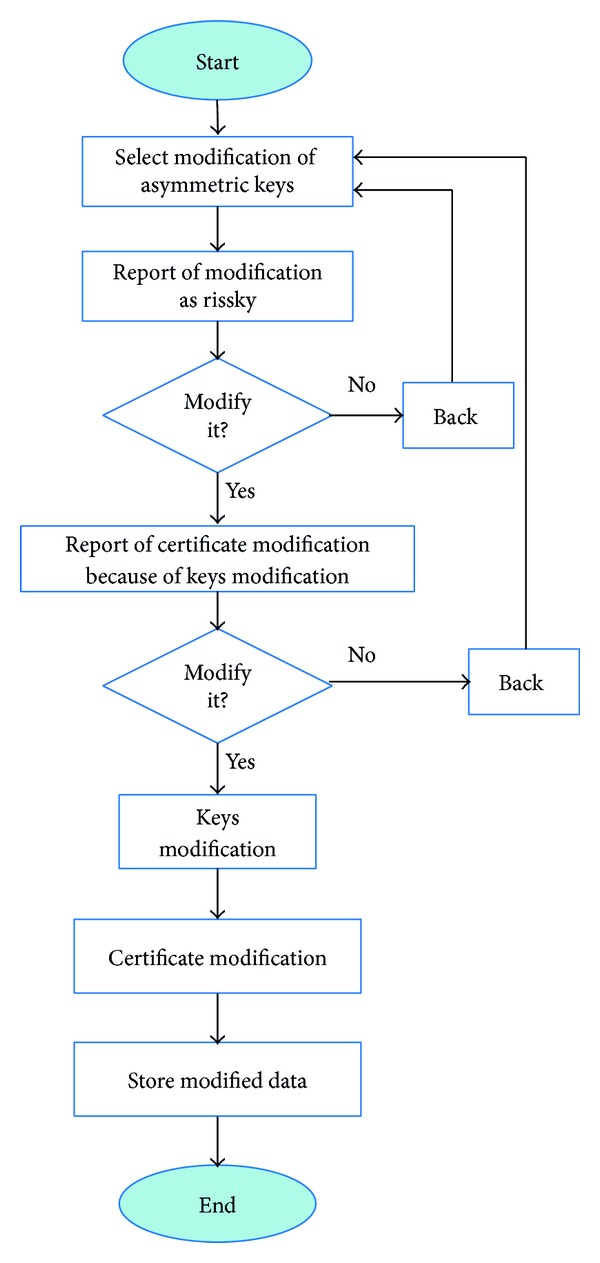
Modification of keys.

When a user decided to modify its asymmetric keys, he is notified of the risks. This notification is shown as a text message in an emergent window. If the user decides to modify it, user's keys are regenerated and the certificate is modified. As Figure 5 shows, after these changes, new data is stored. Finally, the system allows user to stop the process of modification keys before they are changed.

#### 4.2.3. Main Menu

**Figure 6:**
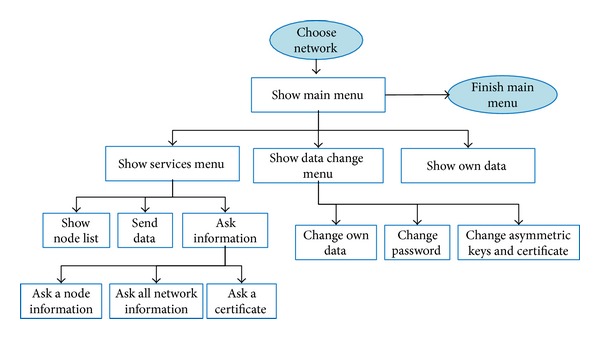
Main menu.

For the development of the software application, we have designed a main menu that includes a submenu the services offered in the mobile cloud computing network, a submenu that allows the user to exchange data, and a submenu to see its own data.  Figure 6 shows all the possibilities that the main menu offers.

#### 4.2.4. Request of Information

In our system, there are two types of information request:

- request for one node: there is a request for one node about specific information;
- request for all nodes: there is a request for all network nodes requesting for the available information in the network (such as shared resources).

**Figure 7:**
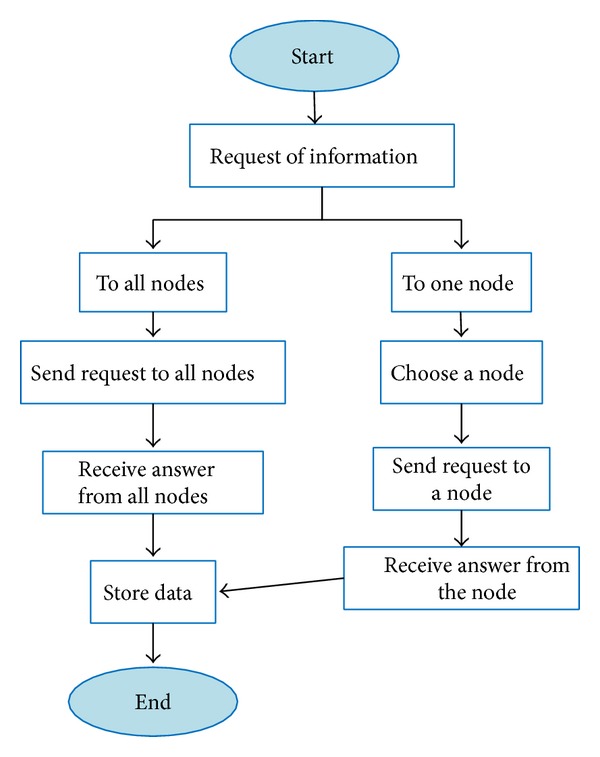
Request of information.

As Figure 7 shows, the information can be requested to one node or to all nodes. After receiving the information requested, these data are stored.

#### 4.2.5. Reply to a Request of Information

**Figure 8:**
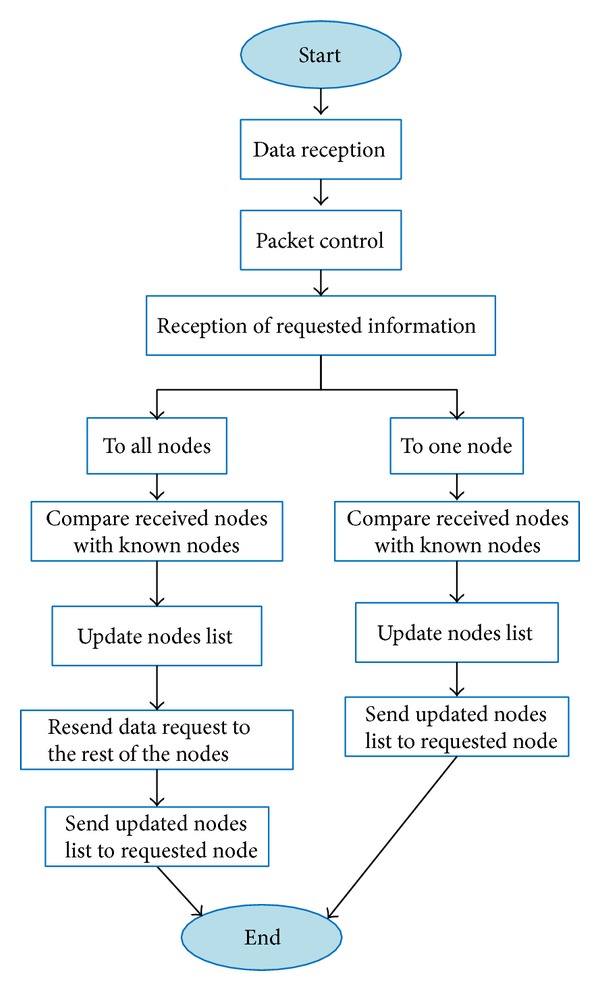
Procedure when information is requested.

Figure 8 shows the process of how to reply to a request of information. When the node receives a request, the reply depends on the type of the received request. If the request is about all network data, the node will reply with the updated data and will forward the request to the rest of the nodes. If the request is just to one node, the receiver node replies with the data request by the source node.

### 4.3. Classes Design

In this subsection, we describe the main designed classes for the proper operation of the spontaneous ad hoc mobile computing network.

#### 4.3.1. Trustednet Ident Class

The* trustednet ident class* (see Algorithm 1) creates an identity card for the node. It contains most of the information about a node and the encryption algorithms that are going to be used by the node. The default constructor generates a timestamp, public key, and a private key.

After the neighbor discovery, the different nodes have to send messages towards their neighbors. These messages contain the identity card of the node. When the neighbor receives this card, it checks if there is nothing changed in the card. This can be checked by calculating the hash of the card. If this new calculated hash is the same as the hash which is included in the message, then nothing is changed.

#### 4.3.2. Trustednet Node Class

It is shown in Algorithm 2. Objects of this class type are nodes. The program/class which uses this class can declare and initialize the node objects. These objects contain a group of neighbors and an identity and the location coordinates.

The* double* ^*∗*^ *coordinates* variable stands for the location coordinates of the node. The* Neighbor* ^∗∗^ *neighbors* variable is an array of pointers that contains the neighbors of the node.* nNeighbors* contains the number of neighbors and* identity* contains the ID card. The method test() generates a new hash out of the data fields and compares it to the SHA-1 hash.

#### 4.3.3. Trustednet Key Class

Trustednet key class has two keys the private and the public one. They are generated when the default constructor is called. The generated keys can have a value up to 999. This is a very basic class. But, it is specially designed for integrating different key generation algorithms.  Algorithm 3 shows the code of this class.

#### 4.3.4. Trustednet Graph Class

Objects of this class type are graphs. It is shown in Algorithm 4. The class which uses this class can declare and initialize graph objects. The graph contains nodes. These nodes are connected by neighbors. The main purpose of this class is to estimate which neighbor it has to send the message to (till the destination receives the message). It performs the routing protocol tasks. The* hash* variable contains a pointer to the network nodes. There is no need to delete a node that does not have neighbors or trusted neighbors because it is a pointer. The* dijkstra* method calculates the path to the nodes it can reach.

Some designed helper methods that are not included before are the following ones. The method onReceiveMessage takes a look at the type of the received message. If this is a public key sending or returning a message, then we check if there was no data loss or change in the identity card. If it is a public key message, then the node sends back a public key return message. When the first node receives a public key return message and there is no data loss or changes, it sets the neighbor as trusted. When there is a broadcasting message, the node calls to the updateNeighborTable method. The method send2NetworkDataPacket is two times declared in the source with different parameters. That happens because the messages for broadcasting are different from those for sending public keys. Mainly they are doing the same. They are setting/getting the data of a trustednet DataPacket message. Afterwards, the node sends the message to the destination node updates neighborTable by using the method updateNeighborTable. These messages are used to discover the neighbor nodes. The table holds an entry for every node in the network if it receives a message from that node. When all the messages are broadcasted, it checks the neighborTable. It will only accept a node as a neighbor if the amount of received messages is above a certain threshold. For instance, we can declare the threshold for every node on 95%, or, in case of 3 nodes in the network, we can declare an independent threshold for each node. So, in this case, node 0 will accept a neighbor if it receives 25% of the broadcast messages.

## 5. Performance Simulation

Castalia 2 is a wireless sensor network simulator based on the OMNeT++ 3 platform [35]. It can be used by developers and researchers who want to test their algorithms and protocols with a realistic node behavior and wireless channel radio model. It is very important to perform the most accurate channel characterization in order to reproduce the system operation in real environments [36]. It can also be used to evaluate different platform characteristics for specific applications. Because it is highly tunable and it can simulate a wide range of platforms. The main features of Castalia are advanced channel/radio model based on empirically measured data, detailed state transition for the radio, highly flexible physical process model, sensing device noise, bias and power consumption, node clock drift and CPU power consumption, and resource monitoring, allowing the design of medium access control protocols with a large number of parameters to tune. Castalia lets us easily implement and import our designed algorithms and protocols while making use of the features provided by the simulator. The modularity, reliability, and speed of Castalia are partly enabled by OMNeT++, which is an excellent framework for building event-driven simulators.

### 5.1. Simulation Parameters

Castalia simulator uses objects from OMNeT++. In Castalia, each model has 3 different methods: initialize(), handle message(), and finish() methods. They are called in this sequence for every single node. In the next subparagraphs, we will talk about different Castalia modules and how to configure them. Each parameter of these modules has a different meaning. All these parameters are initialized in different files. The channel model used is the log shadowing wireless channel model which gives the power loss in dB, given the distance of two nodes *d* and a few parameters. Based on the power loss and the transmission power of a transmitter, we can calculate the power of the signal received at a receiver. By knowing the noise or interference at this receiver, we can calculate the signal to noise ratio or signal to interference ratio, SNR or SIR. Castalia allows us to dynamically calculate the interference from different transmitting nodes and thus dynamically calculate the SNR's or SIR's and the resulting packet reception probabilities.

The radio module tries to capture many features of a real generic low power radio, which is used in wireless sensor network platforms. The following parameters like noise, Bandwidth, modulationType, and encodingType affect the probability of reception. Another parameter is noise floor, which depends on temperature and bandwidth. The receiverSensitivity gives the sensitivity of the receiver. Other parameters are rxPower, listenPower, and sleepPower or transmission parameters like txPowerLevels and txPowerConsumptionPerLevel.

There is a separate module for the medium access control. There are different interesting parameters. The dutyCycle parameter is the fraction of the time that a node listens to the channel. The listenInterval is the time the node stays on listening. Knowing the duty cycle, we can then define the amount of time the node sleeps. If a node is sleeping, then the BeaconIntervalFraction lets us wake up this node. BackOffType let us put the transmission back-off for some time if the channel is not clear and puts the radio to sleep mode.

Castalia lets us work in two models, linear model and nonlinear model. In a linear model, there are 3 different states: (1) a node can be impossible to reach because of a not trusted state, (2) a node can be directly reached if the destination is the node itself or a direct neighbor of the node, (3) and a node has to bypass a message towards another node if the destination can be reached through a neighbor. The nonlinear model uses the Castalia built-in generator. There are three deployments: uniform random deployment, grid deployment, and randomized grid deployment (grid + noise).

### 5.2. Performance Results

Because we want the most accurate results with large number of nodes, we ran the model several times and we provide the most important results in the wireless channel and the MAC layer parameters.

#### 5.2.1. Wireless Channel

This section discusses what happens with our result of our model when we adjust the most important parameters of the wireless channel.

**Figure 9:**
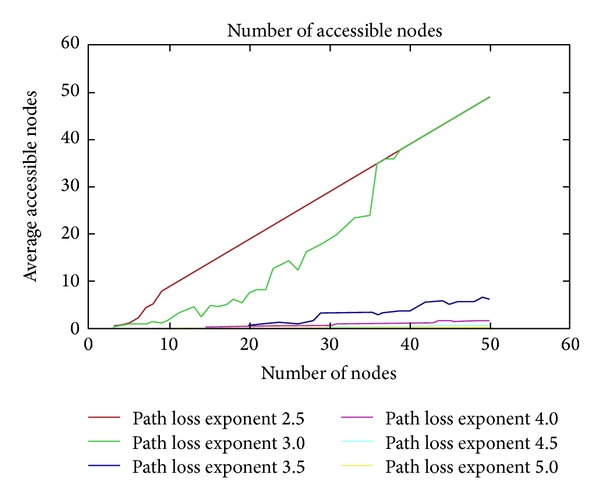
Path loss exponent.

We use path loss exponent of a transmitter to calculate the power of the received signal.  Figure 9 shows the number of the average accessible nodes as a function of number of nodes using different path loss exponent. This simulation is tested in a 100 m by 100 m area. As we know, if the path loss exponent decreases, the power of the signal received at the receiver increases. Taking into account this fact, we can see that the lowest path loss exponent presents the highest average number of accessible nodes. However, the highest path loss exponent shows very small average number of accessible nodes.

**Figure 10:**
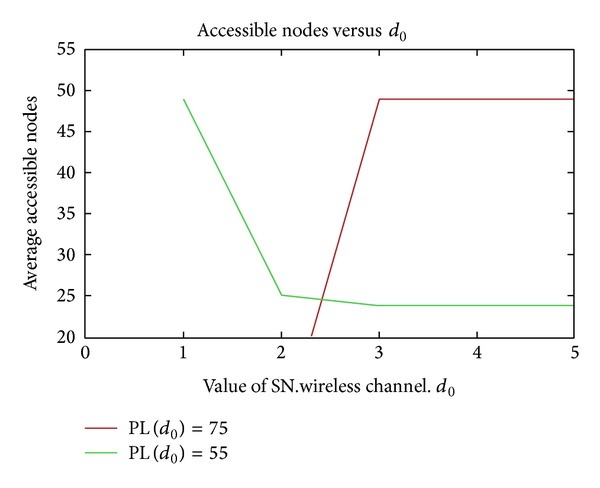
Reference distance.

The PL *d*_0_ is the known path loss at a reference distance *d*_0_. It let us know the initial signal power for each node, also called equivalent isotropically radiated power (EIRP) or, alternatively, effective isotropically radiated power. For the following test, we used PL *d*_0_ = 55 dBm and 75 dBm (which simulates nodes with different transmission power or different antenna gains). The test increases the reference distance with the same known path loss. We used 50 nodes in an area of 100 m by 100 m. Our results are shown in Figure 10.

#### 5.2.2. Medium Access Control

**Figure 11:**
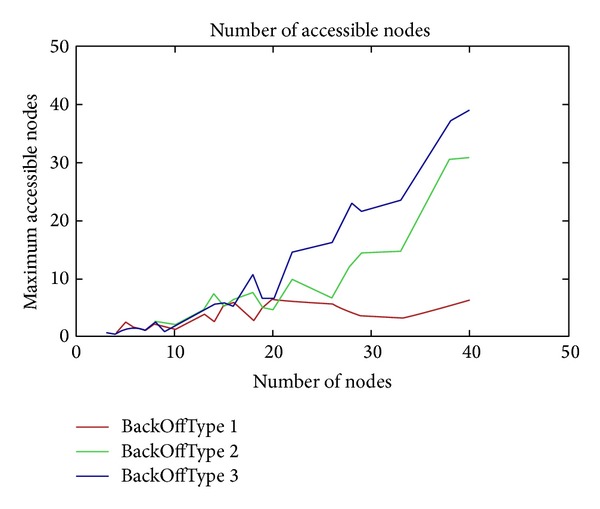
Maximum accessible nodes as a function of the number of nodes.

The parameter BackOffType has a close connection with carrier sensing. It means that before a node starts to transmit a message and before it starts to transmit potential beacons, it checks with the radio to see if the channel is clear. If the channel is not clear, the node has to back off for some time. We have different backOffTypes (Figure 11). Using value 1, the back-off time is constant and it is defined by the BackoffBaseValue parameter. Using value 2, the back-off time has a multiplying value, for example, 1∗*a*, 2∗*a*, 3∗*a*, 4∗*a*,…. We back off for (BackoffBaseValue) ∗ (times). Using a value that is equal to 3, the back-off time is an exponential value (e.g., 2, 4, 8, 16, 32,…). As we can see in Figure 11, the exponential value is the better than the multiplying value and the constant value. We used the additive interference model to simulate this behavior.

**Figure 12:**
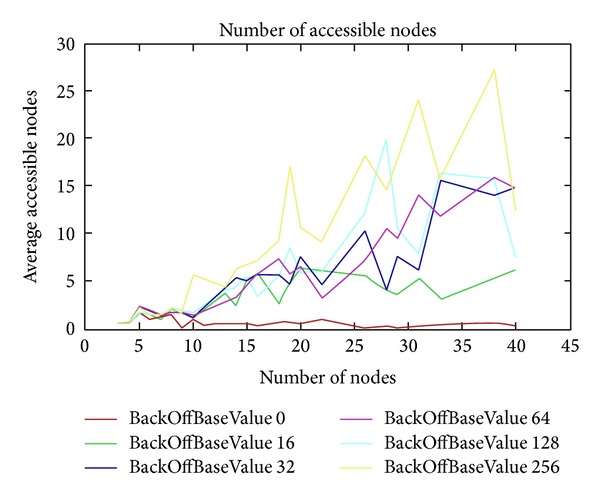
BackOffType = 1.

In the following test, we increase the BackOffBaseValue parameter and look at the difference between the backOff types. For all these tests, we used the additive interference model to simulate this behavior.  Figure 12 shows different BackOffBaseValues (from 0 to 256) for BackOffType 1. Meanwhile, there are not too much difference between BackOffBaseValue 32 and BackOffBaseValue 64, and there is a clear difference between BackOffBaseValue 128 and BackOffBaseValue 256, although we can find some peaks in BackOffBaseValue 128 which have higher values than BackOffBaseValue 256.

**Figure 13:**
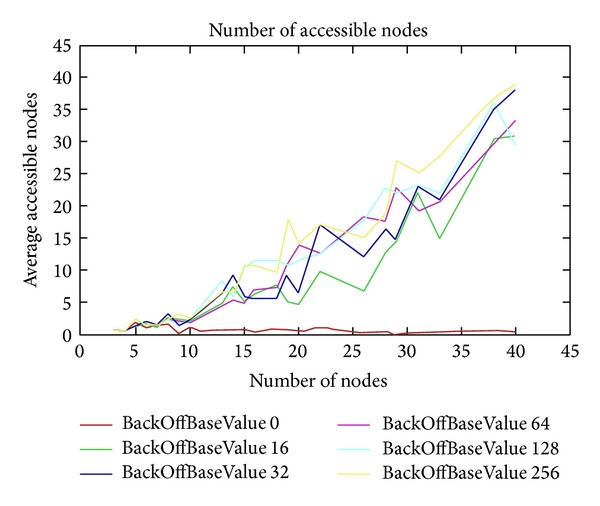
BackOffType = 2.

Figure 13 shows different BackOffBaseValues (from 0 to 256) for BackOffType 2. We can see that except BackOffBaseValue 0, the rest tend to have similar average accessible nodes when the number of nodes increases. The values obtained in this case for the average accessible nodes are higher than those for BackOffType 1.

**Figure 14:**
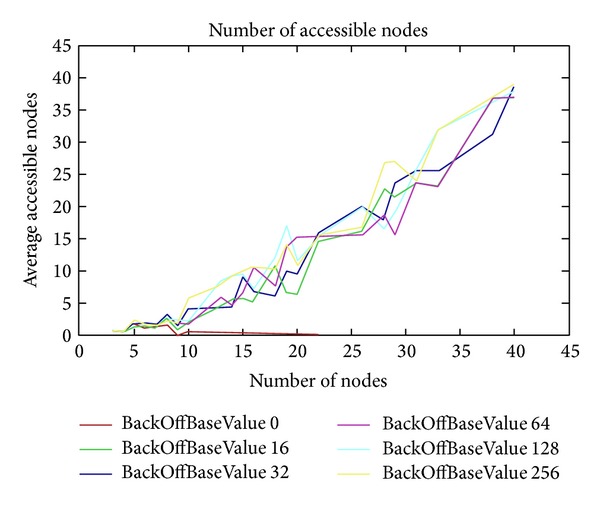
BackOffType = 3.

Figure 14 shows the obtained graphs for different BackOffBaseValues (from 0 to 256) for BackOffType 3. We obtain similar average accessible node values than for BackOffType 2, but in this case the tend of the graphs of BackOffBaseValues are parallel when the number of nodes increases (except for BackOffBaseValue 0).

We have observed that if the BackOffBaseValue gets very high, there is almost no difference between the number of neighbors. But when the BackOffBaseValue is low, for instance, between 0 and 16, then there is a big difference between the amount of neighbors.

**Figure 15:**
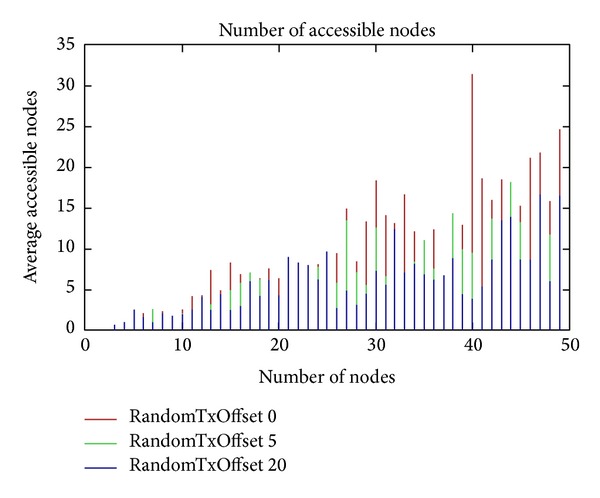
RandomTxOffset.

Castalia uses a random time offset when a node decides to transmit something instead of transmitting it immediately (because it helps to avoid collisions). Now, we made a test of the randomTxOffset parameter. We used again the additive interference model. We observed that the randomTxOffset parameter scores better with a value of 0 than in higher randomTxOffset.  Figure 15 shows the results of our test.

## 6. Deployment 

A prototype to simulate the creation of a virtual cloud computing platform using a spontaneous network has been developed. When the network is created, users can update the network information, sharing resources and services, by asking other nodes. They can ask all network nodes or just one specific node. The receiver node reply to the request with the information requested or it can decide not to share and deny the request.

**Figure 16:**
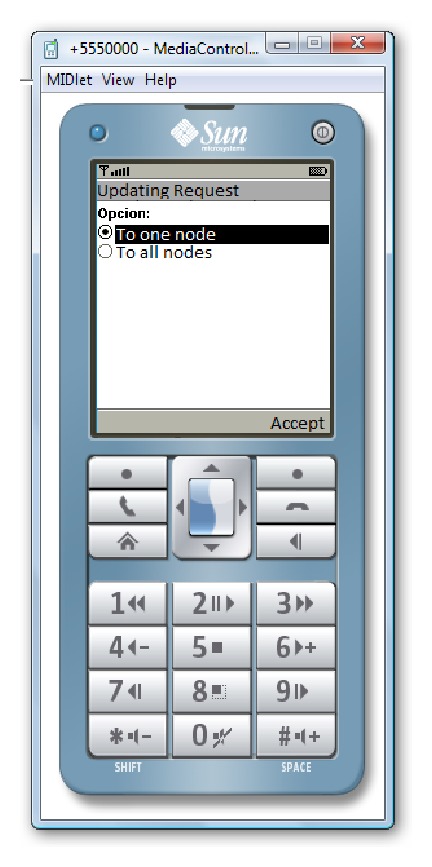
Request menu.

**Figure 17:**
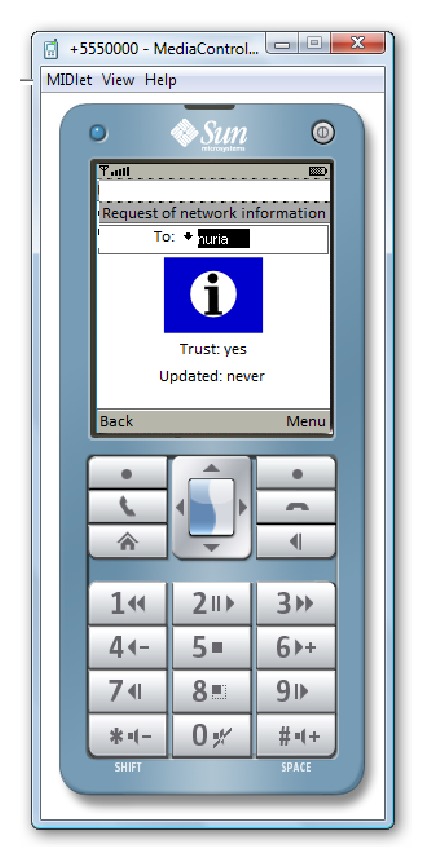
Image in the mobile when there is a request to one node.

Figures 16 and 17 show the designed windows to request some network information. A user can decide to request the data only for a specific network node or for all network nodes. If the user selects to request the data for one node, the user must choose the node to request the data. When a node receives the request, it can decide to reply or not to the request.

**Figure 18:**
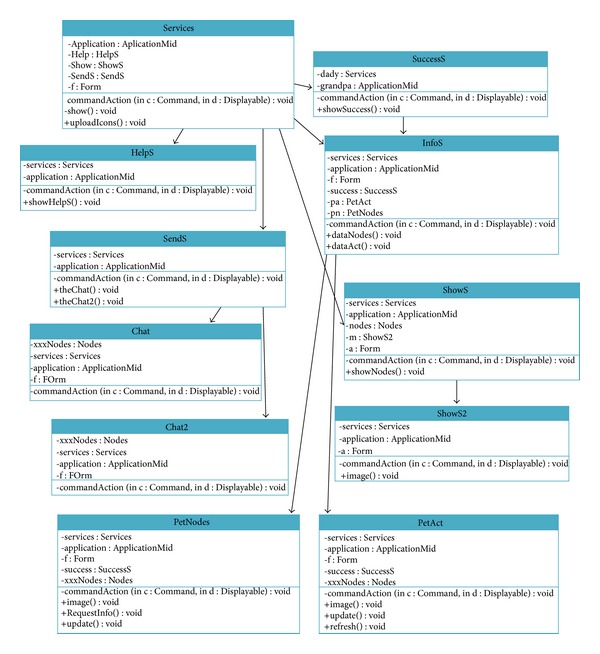
Request diagram class.

Figure 18 shows the diagram class of the request process. HelpS class helps the user with the use of the services menu. SuccessS class informs the user if the process has been processed successfully or if there has been a failure. SendS class offers the user the option to choose between sending the request to one node or to all network nodes. Chat Class lets the user send messages to a node. Chat2 Class lets the user send messages to all network nodes. ShowS Class shows the user the list of the trusted network nodes. ShowS2 class shows the detailed data of one node. InfoS class offers the user a menu to choose a request. PetNodes Class manages the information request about all network nodes. PetAct class manages the information request of one network node.

## 7. Conclusion

Mobile cloud computing networks allow mobile users to share computing resources and applications. In this paper, we proposed a trusted algorithm for creating spontaneous ad hoc mobile cloud computing network. We have developed and tested some algorithms that allow managing the nodes that join and leave the spontaneous ad hoc network. In order to guarantee the network security and the reliability of the communications and transmitted data, we have also developed a trusted algorithm. This algorithm is based on the advanced encryption standard (AES) algorithm and it has implemented a symmetric encryption scheme with simple key management features. We have also deployed the communication protocol procedures and the designed classes. Finally, using Castalia simulator in the OMNeT++ 3 platform, we have implemented a prototype to simulate the creation of a mobile cloud computing system using a spontaneous ad hoc network.

From our results, we can see that, in some cases, as the number of nodes in network increases, the network performance is slightly reduced. However, there are combinations of parameters that maintain the performance level in very promising values.

As future work, we would like to include secure processes based on trust mechanisms and analyze the delay of the secure procedures (proposed in our system) versus the procedures without security systems. Moreover, we will compare the simulation results with real values. In future works, we will test our system in unsecure public cloud environments [37].

## Figures and Tables

**Algorithm 1:**
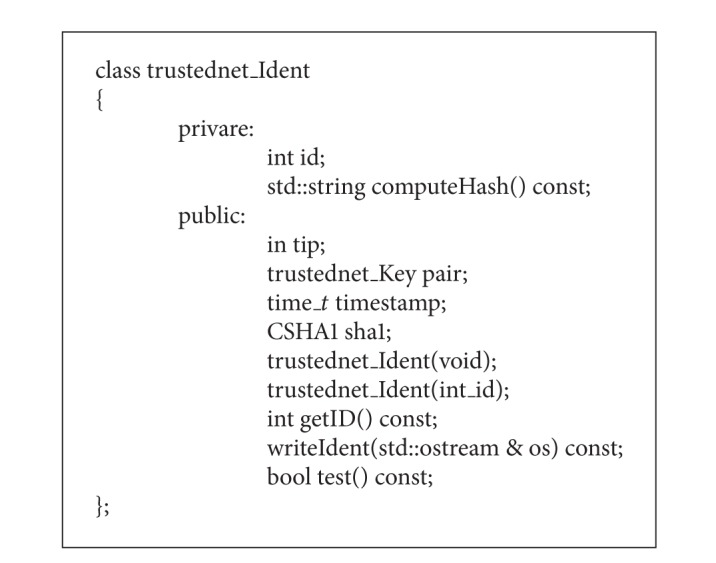
Program code of the class trustednet ident.

**Algorithm 2:**
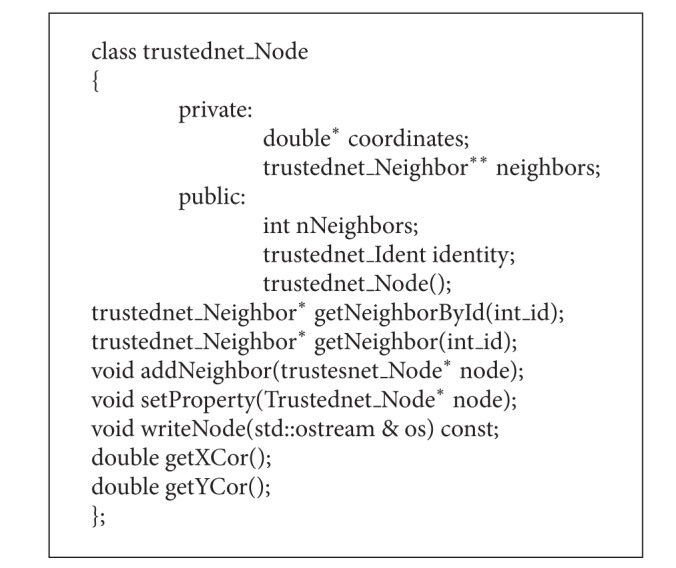
Program code of the class trustednet node.

**Algorithm 3:**
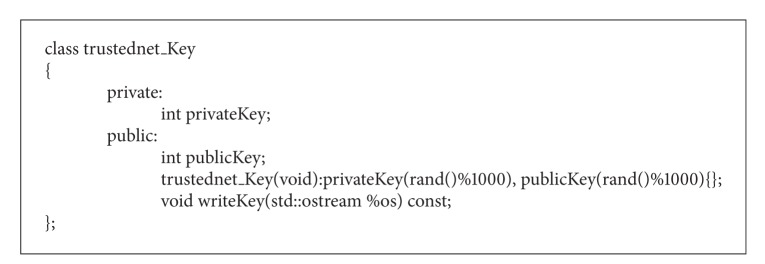
Program code of the class trustednet key.

**Algorithm 4:**
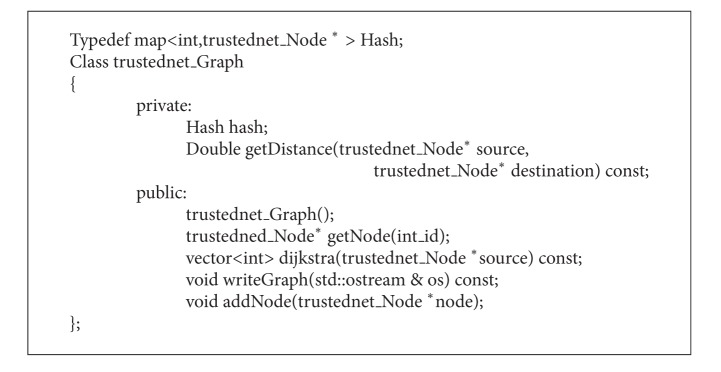
Program code of the class trustednet graph.

**Table 1:** Trust and validity values.

Parameter	Level	When does it happen?
Trust	0	(i) There has been a greeting process between users, but the one in the network does not trust him (ii) The user has reduced the level of confidence to the other one
	1	(i) It has been a greeting process between user and the one in the network that trusts him (ii) The user has increased the level of confidence to the other one

Validity	0	(i) It is not obtained from the greeting process with that user (ii) The validity has not been obtained through a trusted node
	1	(i) It has been obtained directly from the greeting process with that user (ii) It has been obtained through a trusted node
